# Benign Hyperostosis of the Rib

**DOI:** 10.5334/jbsr.3550

**Published:** 2024-03-18

**Authors:** Michiel Van Elsen, Filip M. Vanhoenacker, Annemiek Snoeckx

**Affiliations:** 1 Department of Radiology, University Hospital of Antwerp, Antwerp, Belgium; 2 Department of Radiology, University Hospital of Antwerp, Antwerp, Belgium; Department of Radiology, AZ Sint-Maarten, Mechelen, Belgium; Faculty of Medicine and Health Sciences, UGent/ University Antwerp, Belgium; 3 Department of Radiology, University Hospital of Antwerp, Antwerp, Belgium

**Keywords:** Hyperostosis, diffuse idiopathic skeletal hyperostosis, ribs, benign

## Abstract

*Teaching point:* Benign hyperostosis of the rib is a benign entity consisting of a stress phenomenon that should not be confused with Paget, fibrous dysplasia, or osteoblastic metastasis.

## Case

**Figure 1:**
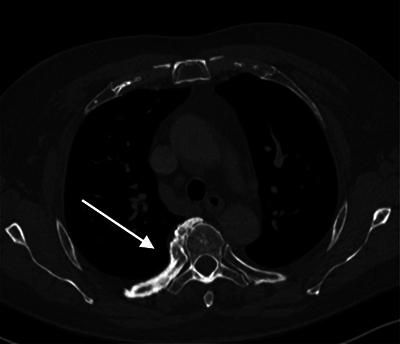
CT thorax axial images in bone window.

A 77-year-old patient underwent a computed tomography (CT) scan of the chest for a 1-year follow-up of a lung nodule, which was unchanged. In addition, there was diffuse cortical thickening of the posterior aspect of the right fifth rib with a large osseous excrescence at the fifth costovertebral joint (Figure 1). The left side was normal.

The increased density of the involved rib was also clearly appreciated on the axial and coronal minimal intensity projection (MIP) images (Figure 2A and 2B).

**Figure 2A:**
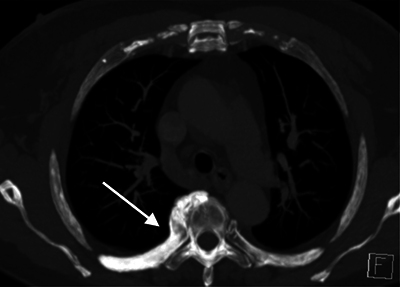
CT thorax axial MIP images.

**Figure 2B:**
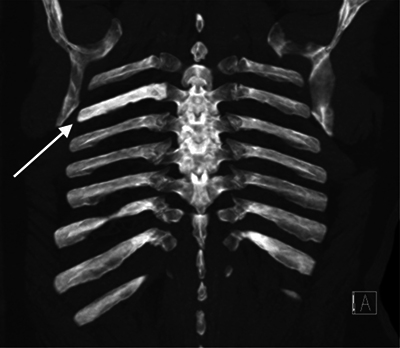
CT thorax coronal MIP images.

**Figure 3:**
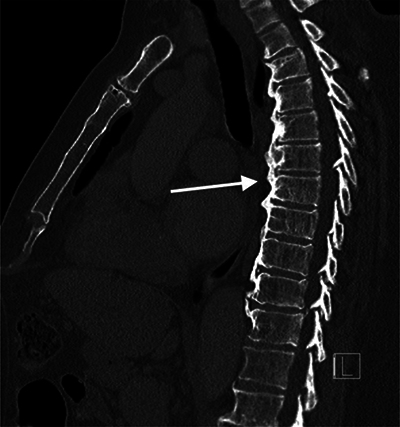
CT thorax sagittal images in bone window.

Sagittal images showed diffuse idiopathic skeletal hyperostosis (DISH) with flowing osteophytes (Figure 3).

## Comments

Benign rib hyperostosis is a benign stress reaction that may be misinterpreted, especially in patients with an oncologic history. It is often seen in correlation with disorders causing excessive vertebral ossification, such as DISH, seronegative spondyloarthritis, and quadriplegia [1].

Hyperostosis typically manifests in the posteromedial aspect of the rib and is commonly observed in conjunction with an osseous excrescence spanning the respective costovertebral joint and associated ossification of the radiate ligament, which attaches the head of the rib to the vertebral body. It is believed that ankylosis of the costovertebral joint causes increased loading forces on the rib due to loss of costovertebral joint mobility, resulting in bony remodeling, and reactive hyperostosis on conventional radiography (CR) and CT. Bone scintigraphy may show increased radiotracer uptake.

Due to aortic pulsations inhibiting ossification, there is a 9:1 predilection for right-to-left rib involvement of the thoracolumbar spine.

The differential diagnosis includes fibrous dysplasia, Paget’s disease, melorheostosis, and renal osteodystrophy. Fibrous dysplasia may lead to fusiform enlargement of the ribs with loss of normal trabeculation and cortical thinning as opposed to thickening. Paget’s disease rarely involves the ribs (1%–4%), showing cortical thickening and bone enlargement, resembling reactive hyperostosis. However, the trabeculae are thickened, and imaging manifestations of Paget’s disease are usually present elsewhere in the skeleton, although monostotic involvement may occur. Cortical thickening in melorheostosis is more extensive and has a typical candle drip appearance with typically associated medullary sclerosis and soft tissue calcifications. Renal osteodystrophy is characterized by generalized bone sclerosis with an associated rugger-jersey spine rather than being isolated.
